# 

**Published:** 1852-01

**Authors:** 


We take great pleasure in publishing the following, which explains itself:

ADAIRVILLE, Ky., Jan. 3d, 1852.

*To the Publishers of the Western Journal of Medicine and Surgery—*

Gentlemen:—Your notice at the end of the last number of the Journal, has been duly observed. I am sorry you regarded my note of the 19th of Nov. as having any reference to yourselves. It was not so intended by me, but on the other hand was designed for (to me) the unknown agent who had assumed (as I thought) the importance of sending me the two duns which I returned to *him*. These two duns came to the office at the *sume time—both* bearing the same date and signature. I thought *one at a time* sufficient, and therefore returned thbm to *him*, with the note which forms the subject of your comment.

So I now address *you*, and enclose you $40, $35 of which you will enter to my credit, and $5 for the Journal of the present year.

Resprctfully,

J. M. JONES.

### RECEIPTS OF MEDICAL JOURNAL FOR DEC. 1851.

Dr. G. W. Forman, High Grove, Ky., ........................ $5 00
M. Hoag, Lodi, Ohio ........................ 5 00
J. Logue, Rural Hill, Tenn ........................ 10 00
J. Wright, Rural Hill, Tenn ........................ 10 00
J. W. Wright, Macon, Tenn ........................ 5 00
W. Boyle, Oakland, Miss. ........................ 10 00
J. S. Lane, Clintonville, Ky. ........................ 5 00
—— Turner, Saloma, Ky. ........................ 5 00
J. M. Jones, Adairville, Ky. ........................ 40 00
P. Stark, Haysville, Ky. ........................ 5 00
T. B. Johnson, Fredonia, Ky ........................ 5 00
R. C. Moore, Bellville, Ind. ........................ 5 00
W. G. Rodes, Rocky Hill, Ky. ........................ 5 00

